# Socio-ecological predictors of participation and dropout in organised sports during childhood

**DOI:** 10.1186/1479-5868-11-62

**Published:** 2014-05-13

**Authors:** Stewart A Vella, Dylan P Cliff, Anthony D Okely

**Affiliations:** 1 Early Start Research Institute, Faculty of Social Sciences, Northfields Avenue, University of Wollongong, Wollongong 2522, Australia

**Keywords:** Youth sport, Attrition, Physical activity, Public health

## Abstract

**Background:**

The purpose of this study was to explore the socio-ecological determinants of participation and dropout in organised sports in a nationally-representative sample of Australian children.

**Methods:**

Data were drawn from Waves 3 and 4 of the Longitudinal Study of Australian Children. In total, 4042 children aged 8.25 (SD = 0.44) years at baseline were included, with 24-months between Waves. Socio-ecological predictors were reported by parents and teachers, while cognitive and health measures were assessed by trained professionals. All predictors were assessed at age 8, and used to predict participation and dropout by age 10.

**Results:**

Seven variables at age 8 were shown to positively predict participation in organised sports at age 10. These included: sex (boy); fewer people in household; higher household income; main language spoken at home (English); higher parental education; child taken to a sporting event; and, access to a specialist PE teacher during primary school. Four variables predicted dropout from organised sports by age 10: lower household income; main language spoken at home (non-English); lower parental education; and, child not taken to a sporting event.

**Conclusions:**

The interplay between child sex, socioeconomic indicators, and parental support is important in predicting children’s participation in organised sports. Multilevel and multicomponent interventions to promote participation and prevent dropout should be underpinned by the Socio-Ecological Model and targeted to high risk populations using multiple levels of risk.

## Background

Participation in organised sports during childhood and adolescence has important benefits for physical, psychological, and social health [1,2]. Pertinent outcomes include higher rates of physical fitness [3], greater involvement in physical activity over time [4,5] lower rates of sedentary behaviour [6], higher rates of self-esteem, more positive social interactions, decreased levels of depressive symptoms [2], and lower rates of obesity [7]. These important benefits may be underpinned by the substantial contribution that participation in organised sports makes to overall levels of physical activity during childhood and adolescence [8-10]. However, there is some evidence to suggest that the health benefits of participation in organised sport go beyond those attributable to physical activity and may be underpinned by the social nature of sports participation. This is consistent with the greater health benefits associated with team sports over individual sports [2].

Given the high prevalence of sports participation in developed countries such as Australia [11], the health benefits of participation in organised sports are potentially significant at a national level. As a consequence, the alarmingly high rate of dropout from organised sports may also be of significance at the population level. Although two-thirds of all Australian children and adolescents participate in organised sports in any given year [11], participation rates start to decline in late childhood and continue to decline with age [12,13]. The statistics on dropout are ambiguous, however, it has been estimated that up to 35% of youth sport participants drop out of organised sports every year. This figure would also include those participants who transfer between sports, however, it has typically been very hard to distinguish between the two and as such the real prevalence and predictors of dropout are unknown [14]. Nonetheless, the high level of dropout throughout late childhood and early adolescence is associated with corresponding declines in physical activity [13], and increasingly sedentary lifestyles [15].

In order to maximise the public health benefits of organised sports participation it is necessary to understand the determinants of both participation and dropout in organised sports. For example, research has shown that Australian girls of a low socioeconomic position experience a disproportionately high number of barriers to sport participation when compared with their peers [16]. The Socio-Ecological Model has been used as a framework to examine the predictors of children’s health and health behaviours [17-19]. According to this model the predictors of sport participation are multidimensional in nature and operate across individual, interpersonal, community and societal levels [19,20]. Eime and colleagues have developed a conceptual model of Health through Sport [2] whereby socio-ecological constructs at all levels predict participation in organised sports. For example, children with strong physical and social functioning, strong parental support for sport, or access to affordable sports programs may be more likely to participate in organised sports. In turn, participation in organised sports has benefits for physical, psychological, and social health. These health outcomes subsequently feed back into the Socio-Ecological Model as determinants of participation in organised sports, whereby higher rates of health predispose young people to maintain sports participation. For example, sports participation leads to greater levels of physical activity and the development of social skills [5,21], which in turn predispose a child to continuing participation in sports. This model provides a theoretical foundation for the study of participation and dropout in organised sports as it predicts that both social (e.g., parental support) and individual (e.g., adiposity) health-related variables derived from the Socio-Ecological Model will underpin an individual’s participation in organised sports.

The purpose of this study was to explore the socio-ecological determinants of participation and dropout in organised sports in a nationally-representative sample of Australian children. Data from the Longitudinal Study of Australian Children offer a unique opportunity to examine the true predictors of dropout from organised sports by distinguishing between those who had transferred between sports and those who had dropped out. Furthermore, it is important to simultaneously investigate the impact of variables at multiple levels of the Socio-Ecological Model in order to account for the multidimensional influences that interact to determine participation in organised sports. Such analyses are important during childhood as they represent a substantial opportunity to understand the factors which may underpin participation in organised sports at a time prior to the onset of high rates of dropout, and where targeted interventions and public policy may be most beneficial. Importantly, constructs informed by the Conceptual Model of Health through Sport as well as Socio-Ecological Models of childhood obesity and health behaviour are important areas of investigation because these health-related variables are purported to predict children’s participation in organised sports [18,19]. This includes individual, interpersonal, community, and societal level variables.

## Method

### Study design and participants

Data were obtained from the Kindergarten (K) cohort of the Longitudinal Study of Australian Children (LSAC) [22]. LSAC is a longitudinal examination of the social, environmental and economic impacts on development and wellbeing in a nationally-representative sample of children. A two-stage sampling design was used whereby Australian postal codes were the primary sampling unit and were stratified by state location (rural/urban). In the second stage, children were randomly selected from the Medicare database which is a national health insurance database and is the most comprehensive database of the Australian population. Wave 1 of data was collected in 2004, and data have been collected every two years following. Trained professionals use face-to-face interviews with the children’s primary parent (in more than 96% of cases this is the child’s mother), parental self-report questionnaires, parent-reported time-use diaries, and teacher self-report questionnaires to collect the range of data. For the K-Cohort, there were 4,983 children aged 4-5 included at Wave 1. This represented a total response rate of approximately 50%. The current study used data obtained from the K-Cohort of LSAC during Waves 3 (2008) and 4 (2010), when information on the children’s sport participation was first collected. Thus, it wasn’t possible to assess sports participation or drop out prior to Wave 3. In total, 4,164 children were included in Wave 4, representing an attrition rate of 16.4% across the 4 Waves. However, only children who had complete sports participation data were included in the study. This resulted in a total of 4,042 children from both Waves 3 and 4 when they were aged 8-9 and 10-11 years, respectively. This represents 97% of total available cases. Ethics approval for the LSAC study was given by the Australian Institute of Family Studies Ethics Committee.

### Measures

#### Sports Participation

Sports participation was measured using two items from the LSAC parent-report questionnaire pertaining to their child’s regular participation in team and individual sports at both Wave 3 and Wave 4. Parents were asked “In the last 12 months, has (your) child regularly participated in team sport (e.g. football, cricket or netball)?”, and, “In the last 12 months, has (your) child regularly participated in individual sport (e.g. tennis, karate or gymnastics)?” In order to qualify as participating ‘regularly’, children were required to have participated at least once per week for a duration of three months or more (e.g., a sporting season). Parents could answer either “yes” or “no” for each item. Children were defined as participating in sports if parents answered “yes” to at least one of the items.

### Predictor variables

Variables used as potential predictors of participation and dropout in organised sports are shown in Table 1. An extensive number of variables were selected according to the Socio-Ecological models presented by Davison and Birch [18], Sallis and Owen [19], and Eime et al. [2]. Individual level variables included measures of physical (e.g., BMI, waist circumference), social (e.g., social functioning), and emotional health (e.g., emotional functioning, mental health), as dictated by the Conceptual Model of Health through Sport. In addition, individual level health behaviours which may displace (e.g., sedentary behaviours) or promote (e.g., physical activity) sports participation, and measures of children’s temperament (e.g., sociability, persistence, temper) which may predispose children to participate or dropout in organised sports were also included. Interpersonal variables included measures of parental support for physical activity and sport (e.g., whether parents took the child to a sporting event, or whether parents were physically active with the child), as well as relevant parenting styles which may underpin a child’s participation (e.g., warm parenting) or dropout (e.g., harsh parenting). Relevant demographic variables which have been shown to be associated with sports participation at the community level (e.g., cultural background, household income, parental education) may also be important predictors and were included [6]. Finally, relevant societal level variables such as measures of societal infrastructure (e.g., access to parks and playgrounds, neighbourhood safety) and school support (e.g., access to specialist physical education teachers and time spent in physical education) may also promote and facilitate participation or dropout in organised sports and were also included. Information presented in Table 1 gives details of the measure that was used, number of items, response format, source of report, and reliability and validity information where appropriate and available. All predictor variables were assessed at Wave 3 when participants were aged 8/9 for their ability to predict sports participation over the subsequent two year period.

**Table 1:** Measures used as socio-ecological predictors of organised sports participation and dropout

**Predictor (Source of data)**	**Description**
**Child characteristics and intrapersonal predictors**	
Child’s Sex (PP)	Child sex (Male/Female)
Indigenous Status (PP)	Aboriginal or Torres Strait Islander (Yes/No)
BMI (TP)	Measured by trained professional [Weight(kg)/Height(m)^2^]
Waist Circumference (TP)	Measured by trained professional (cm)
Gross Motor Coordination – Gross Motor Coordination Scale (PP)	3 item scale assessing how well the child can run, jump, and balance on one leg compared to their peers. 1 (*Better than other children*) – 3 (*Not as well as other children*).
Physical Health - PedsQL Physical Health Subscale (PP)	8 items assessing physical health-related quality of life. 1 (*Never*) to 5 (*Almost always*) [23].
Social Functioning - PedsQL Social Functioning Subscale (PP)	5 items assessing health-related social functioning. 1 (*Never*) to 5 (*Almost always*) [23].
Emotional Functioning - PedsQL Emotional Functioning Subscale (PP)	5 items assessing health-related emotional functioning. 1 (*Never*) to 5 (*Almost always*) [23].
School Functioning - PedsQL School Functioning Subscale (PP)	5 items assessing health-related school functioning. 1 (*Never*) to 5 (*Almost always*) [23].
Mental Health - SDQ Total Difficulties Score (PP)	20 items assessing total number of psychological difficulties including peer, emotional, hyperactivity, and conduct problems [24].
Injuries (PP)	1 item: In the last 12 months, how many times did the child need medical attention from a doctor or hospital because he/she was hurt or injured?
Screen Time (weekly) (PP)	Sum of hours watching TV and DVDs, using computer, and playing electronic games on a typical weekday (×5), and on a typical weekend day (×2).
Time in Physical Activity (PP)	Sum of items “Active free play”, “Organised Physical Activity”, “Riding a Bike” and “Walking”, derived from the LSAC Time Use Diary on one random weekday (×5) and weekend day (×2).
PA Level during Organised School PA (Te)	1 item: During organised activities for your class, how does this child compare with other children in the class in terms of level of physical activity? 1 (*A lot less active than most*) to 5 (*A lot more active than most*).
PA Level during School Recess and Lunch (Te)	1 item: During play with friends at recess or lunch time, how does this child compare with other children in the class in terms of level of physical activity? 1 (*A lot less active than most*) to 5 (*A lot more active than most*).
Introverted Temperament - School-Aged Temperament Inventory Introversion subscale (PP)	4 items assessing the child’s initial response to new people and situations. 1 (*Never*) to 5 (*Always*) [25].
Persistent Temperament - School-Aged Temperament Inventory Persistent Subscale (PP)	4 items assessing the degree of self-direction that the child exhibits in fulfilling tasks and other responsibilities. 1 (*Never*) to 5 (*Always*) [25].
Reactive Temperament - School-Aged Temperament Inventory Reactive Subscale (PP)	4 items assessing the intensity and frequency with which the child exhibits negative effect. 1 (*Never*) to 5 (*Always*) [25].
**Parenting styles, family characteristics, and interpersonal predictors**	
Number of People in Household (PP)	Number of people in household
Main Language Spoken at Home (PP)	1 item: What is the main language spoken at home? Categorised as English/Other.
Primary Parent Highest Education (PP)	Primary parent’s highest level of education. 1 (*Not finished high school*) to 3 (*Tertiary degree*).
Standardised Household Income (PP)	Self-reported household income in dollars per week divided by the square root of the number of people in the household [26].
Parental PA – Days per week of moderate to vigorous physical activity (PP)	1 item: About how many days each week do you do at least 30 minutes of moderate or vigorous physical activity (like walking briskly, riding a bike, gardening, tennis, swimming, running, etc?) 0-7.
Parental Concern about Child’s Weight (PP)	1 item: How concerned are you about your child’s weight at the moment? 1 (*Not at all*) to 4 (*Very*).
Parental PA with Child (PP)	In the past week, on how many days have you or an adult in your family, played a game outdoors or exercised together like walking, swimming, cycling? 0 (*Not in the past week*); 1 (*1 or 2 days*); 2 (*3-5 days*); 3 (*6-7 days*).
Child taken to Sporting Event (PP)	In the past month, has child gone to a sporting event where the child was not a player, with you or another family member? Yes/No
Warm Parenting Style (PP)	6 items assessing the frequency of warm and affectionate behaviours towards the child [27].
Harsh Parenting Style (PP)	3 items assessing the frequency of yelling and smacking interactions with the child [28].
Protective Parenting Style - Overprotective parenting scale (PP)	3 items assessing the extent to which the parent is overprotective of the child [29].
Child gets Bullied at School (PP)	In the last 12 months, has study child been bullied at school? (‘At school’ includes travel to and from school). Yes/No
**Community, demographic, and social predictors**	
Neighbourhood Remoteness - Australian Remoteness Indicator for Areas Categories (PP)	Australian Remoteness Indicator for Areas Categories. 0 (*Highly accessible*) to 4 (*Very remote*) [30].
Availability of Parks and Playgrounds (PP)	1 item: There are good parks, playgrounds and play spaces in this neighbourhood. 1 (*Strongly agree*) to 4 (*Strongly disagree*).
Availability of Public Transport (PP)	1 item: There is access to close, affordable, regular public transport in this neighbourhood. 1 (*Strongly agree*) to 4 (*Strongly disagree*).
Safe to Play Outside (PP)	1 Item: How strongly do you agree or disagree with the statement: It is safe for children to play outside during the day. 1 (*Strongly agree*) to 4 (*Strongly disagree*).
Time spent in PE during School Time (Te)	1 Item: How much time per week, in total, do children in your class spend participating in physical education?
Access to a PE Teacher at School (Te)	1 Item: Does this child’s class have access to a specialist Physical education teacher? Yes/No

Note: *PP* Primary Parent, *TP* Trained Professional, *Te* Teacher, *TUD* Time Use Diary, *PA* Physical Activity, *PE* Physical Education, *PedsQL* Pediatric Quality of Life Scale, *SDQ* Strengths and Difficulties Questionnaire.

### Statistical analyses

All data were analysed using IBM SPSS statistical software (version 19, IBM, New York, United States). Selected demographic characteristics and predictor variables were used to summarise the characteristics of the sample, including weight status [31]. We then conducted two separate analyses to examine (i) predictors of dropout in organised sports, and (ii) predictors of participation in organised sports. In order to assess predictors of dropout we examined all children who were participating in sport at age 8 to determine which variables predicted dropout by age 10. In order to assess predictors of participation in organised sports we conducted analyses on all children at age 8 to examine which variables predicted sports participation at age 10. Both analyses followed the same strategy. At the first stage all potential predictor variables were entered individually into an unadjusted binary logistic regression model. Significance at this stage was set at *p* < 0.05. For each comparison pair potential collinearity was assessed amongst all significant predictor variables using the estimated individual variance inflation factor (VIF), tolerance for each predictor, and the mean VIF. Items with a VIF of >10 and a tolerance of <0.1 were to be removed from subsequent analyses [32,33]. Where the mean VIF was significantly greater than 1, but no predictor variable had an individual VIF >10, the variable with the highest VIF was to be removed. However, no collinearity was detected and no items were removed from secondary analyses. In the second stage of analyses all predictor variables were entered into fully adjusted binary logistic regression models in order to examine potentially significant predictors after adjusting for the effect of all other significant predictor variables. The significance level at this stage was set at *p* < 0.05. Lastly, the Hosmer and Lemeshow test was used to assess goodness-of-fit for each of the final binary logistic regression models.

## Results

### Participants

There were 4,164 participants who provided data at Wave 4 of the LSAC study. Of those, a total of 4,042 (97%) had complete sports participation data at both Wave 3 and Wave 4, and were included in analyses. Participants with complete data in Waves 3 and 4 (n = 122) were more likely to have a parent that was older, hold a tertiary education degree, and speak English at home. Complete data were also more likely for non-Indigenous children, children enrolled in Government schools (vs. Independent schools), and in areas where higher proportions of residents had completed high school [34,35]. Selected demographic characteristics of the entire sample and by grouping are shown in Table 2. At age 10, organised sports participants were more likely to be boys, speak English as a main language, were of a higher socioeconomic position (all *p* < .001), and were more physically active (*p* = .011) than nonparticipants. There were no differences according Indigenous status, BMI, or sedentary time. Children who dropped out between the ages of 8 and 10 were more likely to be girls (*p* < .001), non-English speaking (*p* = .002), and Indigenous (*p* = .026), were of a lower socioeconomic position (*p* < .001), and were less physically active at age 8 (*p* = .024). There were no differences according to BMI or sedentary time.

**Table 2:** **Selected demographic characteristics of the sample and by participation group**^**a**^

**Characteristic**	**Group, n (%)**				
	**Entire sample**	**Participants at age 10**	**Non-participants at age 10**	**Dropouts**^**b**^	**Non-dropouts**^**c**^
**Total**	4042	3190 (79)	852 (21)	389 (12)	2777 (88)
**Boys**	2069 (51)	1751 (55)	318 (36)	176 (45)	1575 (57)
**Indigenous**	105 (3)	66 (2)	39 (5)	14 (4)	52 (2)
**English as main language**	3635 (90)	2886 (91)	749 (86)	338 (87)	2548 (92)
**Normal weight**	2855 (71)	2265 (71)	590 (68)	282 (73)	1983 (72)
**Overweight**	695 (17)	538 (17)	157 (18)	52 (13)	486 (18)
**Obese**	243 (6)	171 (5)	72 (8)	27 (7)	144 (5)

^a^Data were drawn from the Longitudinal Study of Australian Children Waves 3 (2008) and 4 (2010) when children were aged 8 and 10 years respectively.

^b^Children who were participating in organised sports at age 8 but no longer participating at age 10.

^c^Children who were participating in organised sports at age 8 and still participating at age 10.

### Unadjusted analyses

Table 3 presents the results of the unadjusted analyses used to assess the significant Socio-Ecological predictors of participation and dropout in organised sports. In total, 24 of the 48 variables entered as potential predictors of participation in organised sports at age 10 were significant in unadjusted analyses and were retained for the fully adjusted model. This included a range of individual, interpersonal/family, and community level predictors. Of note, Indigenous status, BMI, waist circumference, and general health at age 8 did not predict sports participation at age 10 in unadjusted analyses.

**Table 3:** **Socio-Ecological predictors of participation and dropout in organised sports in unadjusted analyses**^**a**^

**Predictor (reference category)**^**b**^	**Odds ratio (95% CI), *p *** **value**	
	**Participation**^**c**^	**Dropout**^**d**^
**Child characteristics and intrapersonal predictors**		
Child’s Sex (female)	2.579 (2.097 – 3.171), *p* = .000*	0.631 (.509 – .781), *p* = .000*
Indigenous Status (Indigenous)	1.633 (.897 – 2.971), *p* = .108	0.512 (.281 – .932), *p* = .029*
BMI	.972 (.939 – 1.007) *p* = .111	0.994 (.955 – 1.034), *p* = .756
Waist Circumference	.998 (.988 – 1.008), *p* = .744	1.002 (.991 – 1.013), *p* = .722
Gross Motor Coordination	.335 (.264 – .426), *p* = .000*	1.836 (1.427 – 2.362), *p* = .000*
Physical Health	1.020 (1.013 – 1.027), *p* = .000*	0.983 (.976 – .991), *p* = .000*
Social Functioning	1.019 (1.013 – 1.024), *p* = .000*	0.986 (.980 – .992), *p* = .000*
Emotional Functioning	1.006 (.999 – 1.012), *p =* .097	0.993 (.986 – 1.000), *p* = .046*
School Functioning	1.018 (1.011 – 1.024), *p* = .000*	0.984 (.977 – .990), *p* = .000*
Mental Health	.933 (.916 -.950), *p* = .000*	1.052 (1.031 – 1.073), *p* = .000*
Injuries	1.340 (1.063 – 1.688), *p* = .013*	0.921 (.745 – 1.137), *p* = .442
Screen Time (weekly)	1.000 (1.000 – 1.000), *p* = .166	1.000 (1.000 – 1.000), *p* = .090
Time in Physical Activity	1.000 (1.000 – 1.001), *p* = .011*	1.000 (.999 – 1.000), *p* = .024*
PA Level during Organised School PA	1.044 (1.023 – 1.064), *p* = .000*	0.973 (.952 – .995), *p* = .014*
PA Level during School Recess and Lunch	1.045 (1.024 – 1.066), *p* = .000*	0.974 (.953 – .996), *p* = .023*
Sociable Temperament	.694 (.603 – .799), *p* = .000*	1.280 (1.100 – 1.489), *p* = .001*
Persistent Temperament	1.117 (.987 – 1.265), *p* = .080	0.916 (.802 – 1.047), *p* = .198
Reactive Temperament	.915 (.795 – 1.053), *p* = .214	1.099 (.945 – 1.278), *p* = .220
**Parenting styles, family characteristics, and interpersonal predictors**		
Number of People in Household	.830 (.764 – .901), *p* = .000*	1.042 (.947 – 1.145), *p* = .401
Main Language Spoken at Home (Non-English)	2.151 (1.623 – 2.851), *p* = .000*	.596 (.431 – .824), *p* = .002*
Primary Parent Highest Education (Tertiary)	.329 (.264 – .409), *p* = .000* (<HS)	1.894 (1.508 – 2.379), *p* = .000* (<HS)
	.559 (.401 – .780), *p* = .001* (HS)	1.415 (1.003 – 1.995) *p* = .048* (HS)
Standardised Household Income	1.001 (1.001 – 1.001), *p* = .000*	0.999 (.999 – .999), *p* = .000*
Parental PA	1.030 (1.007 – 1.053), *p* = .009*	0.972 (.948 – .996), *p* = .021*
Parental Concern about Child’s Weight	.830 (.716 – .963), *p* = .014*	1.1223 (1.044 – 1.433), *p* = .013*
Parental PA with Child	1.395 (1.243 – 1.567), *p* = .000*	0.779 (.687 – .883), *p* = .000*
Child taken to Sporting Event (yes)	.234 (.185 – .294), *p* = .000*	2.557 (2.042 – 3.203)*, p* = .000*
Warm Parenting Style	1.157 (.953 – 1.404), *p* = .142	0.877 (.714 – 1.076), *p* = .208
Harsh Parenting Style	.992 (.919 – 1.070), *p* = .835	0.983 (.904 – 1.068), *p* = .678
Protective Parenting Style	.682 (.584 – .795), *p* = .000*	1.261 (1.070 – 1.486), *p* = .006*
Child gets Bullied at School (yes)	.766 (.622 – .942), *p* = .012*	1.308 (1.046 – 1.635), *p* = .018*
**Community, demographic, and social predictors**		
Neighbourhood Remoteness	.984 (.915 – 1.057), *p* = .659	0.928 (.847 – 1.017), *p* = .112
Availability of Parks and Playgrounds	.753 (.668 – .848), *p* = .000*	1.178 (1.037 – 1.338), *p* = .012*
Availability of Public Transport	.952 (.873 – 1.030), *p* = .264	0.942 (.862 – 1.031), *p* = .193
Safe to Play Outside	.858 (.767 – .960), *p* = .008*	1.182 (1.048 – 1.333), *p* = .007*
Time spent in PE during School Time	1.000 (.998 – 1.001), *p* = .678	0.999 (.998 – 1.001), *p* = .499
Access to a PE Teacher at School (no)	1.385 (1.094 – 1.752), *p* = .007*	0.701 (.546 – .899), *p* = .005*

Note: *HS* High School, *PA* Physical Activity.

*Denotes significant at *p* < .05, and was entered into adjusted analyses.

^a^Data were drawn from the Longitudinal Study of Australian Children Waves 3 (2008) and 4 (2010) when children were aged 8 and 10 years respectively.

^b^All predictors were measured at age 8.

^c^Children who were participating in organised sports at age 10.

^d^Children who were participating in organised sports at age 8 but were no longer participating at age 10.

Twenty-four variables were significantly predictors of dropout from organised sports by age 10 in unadjusted analyses, including a range of individual, interpersonal/family, and community level variables. As with predictors of participation, BMI, waist circumference, and general health were not significant predictors. However, in contrast with participation, Indigenous status was a significant predictor of dropout in unadjusted analyses. All 24 significant predictors were entered into the fully adjusted model.

### Predictors of participation

Results of the adjusted model used to predict participation in organised sports at age 10 are presented in Table 4. In total, 7 of the variables entered were found to predict sports participation at age 10 after adjusting for the effect of all 24 predictors found to be significant in unadjusted analyses. At an individual level, sports participants were more likely to be boys. Measures of physical and mental health did not predict subsequent sports participation, nor did physical activity. At an interpersonal/family level, sports participants were more likely to live in houses with fewer people and with a greater standardised household income, and who took them to sporting events. Sports participants were also more likely to have more highly educated parents, and to speak English as a main language at home. Parent’s level of physical activity and parenting style were not predictive of subsequent sports participation. At a community level, children who had access to a specialist Physical Education teacher at age 8 were more likely to participate in organised sports at age 10. Socioeconomic position and neighbourhood safety were not predictive of subsequent sports participation. Overall, the fully adjusted model proved to be a good predictor of participation in organised sports (*χ*^2^_(8)_ = 9.14, *p* = .331), successfully predicting 82.6% of cases.

**Table 4:** **Socio-ecological predictors of participation and dropout in organised sports in adjusted analyses**^**a**^

**Predictor (reference category)**^**b**^	**Odds ratio (95% CI), *p *** **value**	
	**Participation**^**c**^	**Dropout**^**d**^
**Child characteristics and intrapersonal predictors**		
Child’s Sex (female)	1.742 (1.328 – 2.287), *p* = .000*	0.772 (0.537 – 1.107), *p* = .160
Indigenous Status (Indigenous)	-	1.249 (0.346 – 4.506), *p* = .734
Gross Motor Coordination	0.850 (0.605 – 1.194), *p* = .350	0.952 (0.606 – 1.497), *p* = .833
Physical Health	1.010 (0.999 – 1.021), *p* = .071	0.989 (0.974 – 1.003), *p* = .131
Social Functioning	0.993 (0.983 – 1.004), *p* = .231	1.001 (0.986 – 1.016), *p* = .913
Emotional Functioning	-	1.011 (0.997 – 1.025), *p* = .121
School Functioning	1.004 (0.993 – 1.014), *p* = .509	0.996 (0.982 – 1.010), *p* = .550
Mental Health	0.973 (0.943 – 1.005), *p* = .097	1.033 (0.989 – 1.080), *p* = .145
Injuries	1.260 (0.947 – 1.678), *p* = .113	-
Time in Physical Activity	1.000 (1.000 – 1.000), *p* = .740	1.000 (1.000 – 1.000), *p* = .960
PA Level during Organised School PA	1.154 (0.999 – 1.331), *p* = .051	0.879 (0.732 – 1.056), *p* = .167
PA Level during School Recess and Lunch	1.107 (0.980 – 1.250), *p* = .102	0.911 (0.778 – 1.066), *p* = .245
Sociable Temperament	0.888 (0.748 – 1.056), *p* = .179	1.201 (0.954 – 1.513), *p* = .120
**Parenting styles, family characteristics, and interpersonal predictors**		
Number of People in Household	0.855 (0.762 – 0.958), *p* = .007*	-
Main Language Spoken at Home (Non-English)	1.701 (1.129 – 2.564), *p* = .011*	0.477 (0.278 – 0.820), *p* = .007*
Primary Parent Highest Education (Tertiary)	0.583 (0.436 – 0.778), *p* = .000* (<HS)	2.014 (1.353 – 2.997), *p* = . 001*(<HS)
	0.732 (0.484 – 1.107), *p* = .139 (HS)	1.834 (1.076 – 3.128), *p* = .026* (HS)
Standardised Household Income	1.001 (1.000 – 1.001), *p* = .000*	0.999 (0.999 – 1.000), *p* = .004*
Parental PA	0.971 (0.907 – 1.040), *p* = .401	1.004 (0.914 – 1.104), *p* = .932
Parental Concern about Child’s Weight	1.089 (0.878 – 1.351), *p* = .437	0.989 (0.742 – 1.320), *p* = .943
Parental PA with Child (yes)	0.849 (0.511 – 1.412), *p* = .415	0.887 (0.722 – 1.089), *p* = .251
Child taken to Sporting Event (yes)	0.385 (0.291 – 0.510), *p* = .000*	2.517 (1.734 – 3.653), *p* = .000*
Protective Parenting Style	0.968 (0.806 – 1.162), *p* = .724	1.003 (0.783 – 1.284), *p* = .984
Child gets Bullied at School (yes)	1.090 (0.815 – 1.460), *p* = .560	1.069 (0.712 – 1.605), *p* = .747
**Community, demographic, and social predictors**		
Availability of Parks and Playgrounds	0.884 (0.754 – 1.036), *p* = .128	1.022 (0.821 – 1.272), *p* = .844
Safe to Play Outside	1.000 (0.858 – 1.166), *p* = .997	1.085 (0.881 – 1.336), *p* = .443
Access to a PE Teacher at School (no)	1.353 (1.025 – 1.785), *p* = .033*	0.805 (0.553 – 1.172), *p* = .259

Note: *HS* High School, *PA* Physical Activity.

*Denotes significant at *p* < .05.

^a^Data were drawn from the Longitudinal Study of Australian Children Waves 3 (2008) and 4 (2010) when children were aged 8 and 10 years respectively.

^b^All predictors were measured at age 8.

^c^Children who were participating in organised sports at age 10.

^d^Children who were participating in organised sports at age 8 but were no longer participating at age 10.

### Predictors of dropout

Results of the fully adjusted model used to predict dropout from organised sports between ages 8 and 10 are presented in Table 4. Of the 24 variables entered into the fully adjusted model, 4 predicted dropout from organised sports after adjusting for the effects of all other variables. No individual level variables were found to predict dropout. At the interpersonal/family level, children who dropped out of sport were less likely to have been taken to a sporting event in the last month, resided in a household with a lower standardised income, had parents with lower educational attainment, and were more likely to be non-English speakers at age 8. Notably, child sex, Indigenous status, various physical and mental health measures, and all community level variables were not predictive of dropout from organised sports. Overall, the fully adjusted model proved to be a good predictor of dropout from organised sports (*χ*^2^_(8)_ = 5.41, *p* = .219), and successfully predicted 89.6% of cases.

## Discussion

In this large and nationally representative sample of Australian children, prospective analyses between the ages of 8 and 10 years revealed 7 socio-ecological predictors of participation in organised sports during childhood, and 4 predictors of dropout. Of these, there was a distinct overlap between the predictors of participation and dropout in organised sports where indicators of greater social disadvantage predicted both nonparticipation and dropout in organised sports. This is consistent with previous research which shows that differences in children’s sports participation are best accounted for by sociocultural and socioeconomic indicators [6,36]. This study also found that children being taken to a sporting event at age 8 predicted participation and maintenance of sports participation at age 10. This may provide an indication that, consistent with the correlates of physical activity [37], parental support is an important facilitator of sports participation among children. This may be especially so because parents or caregivers typically play a key role in children’s enrolment and continued participation in organised sports by way of finance, time, and transportation. Parents’ investment in adolescents’ sporting endeavours has been shown be highly predictive of dropout from organised sport [38]. Together, these results show that sports participation during childhood can best be predicted by a combination of socioeconomic, cultural, and parental support variables.

Previous Australian research has shown that socioeconomic variables and parental support may not be independent of one another [16]. Dollman and Lewis found that children of a lower socioeconomic status reported a greater number of barriers to sports participation and lower levels of both instrumental and affective parental support for playing sport than children of a higher socioeconomic status. Furthermore, these differences were more pronounced in girls. This is consistent with the results of the current study where child sex, socioeconomic indicators, and parental support predicted sports participation, and is supported by the results of Toftegaard-Støckel and colleagues [36] who found that socioeconomic and sociocultural factors were the greatest determinants of adolescent’s sport participation in a sample of Danish children. It is possible that there exists an important interplay between child sex, socioeconomic variables, and parental support in the prediction of children’s participation in organised sports. In particular, girls from lower socioeconomic areas who receive little parental support are most at risk of nonparticipation.

The current results reinforce the importance of using the Socio-Ecological Model to study participation and dropout in organised sports because it enables simultaneous adjustment for the influence of factors at multiple levels of influence [20]. It is imperative, therefore, that interventions to promote participation and prevent dropout in organised sports are appropriately targeted at populations who are at highest risk by accounting for risk at multiple levels (individual, familial, and societal). This is important because dropout from organised sports and nonparticipation may have short- and long-term health consequences for children, including higher rates of obesity and physical inactivity [5,7]. This is particularly problematic for children from a low socioeconomic position and minority cultural backgrounds who are already at greater risk of health conditions such as obesity [39,40]. Furthermore, to maximise their effectiveness these interventions should include multiple components aimed at intervening at multiple levels of influence. This may include issues such as access and affordability, parental support, as well as children’s motivation for organised sports. Such factors have been prioritised in both government and non-government reports in Australia which aim is to increase national participation in organised sports and physical activity [41,42].

A particularly novel finding of this study is that children who had access to a specialist physical education teacher at school at the age of 8 were more likely to participate in organised sports at age 10. This was over and above the impact of socioeconomic status, and it is therefore unlikely that this is due to the possibility that schools of a higher socioeconomic status being more likely to employ a specialist physical education teacher. Therefore, it may be that access to a specialist physical education teacher predisposes children to participation in organised sports. For example, specialist physical education teachers in primary schools have been shown to facilitate greater sport-related outcomes such as higher levels of physical activity, greater fitness, and greater levels of fundamental movement skills when compared with untrained teachers [43-45]. Thus, it is possible that access to a specialist physical education teacher can predispose children to participate in organised sports by increasing skills and behaviours that are conducive to successful participation in organised sports.

This study found no evidence to support the hypothesis that physical, social, or psychological health are predictors of organised sports participation during childhood. Given that overweight and obese Australian youth participate in organised sports at the same rate as their normal weight peers [6], it is possible that some Australian children are being enrolled in organised sports to improve their health, and thus, measures of physical health are not predictors of participation. This may also be true for children with lower social or psychological health who may be enrolled in sport in order to gain social or psychological benefits. More research is needed to investigate the directionality of relationships between participation in organised sports and measures of physical, social and psychological health. In particular, potential bi-directionality should be investigated in light of the Conceptual Model of Health through Sport [2] which proposes a feedback loop that operates between the physical, social, and psychological health benefits of sports participation and the likelihood that children will commence or maintain participation in sports.

A strength of this study was to distinguish between children who dropped out of all organised sports and children who transferred between sports, thus allowing the prediction of dropout. To our knowledge, this is the first study to do so. This study also examined a large number of socio-ecological predictors of participation and dropout in organised sports during childhood, thus allowing a comprehensive analysis of potential predictors. However, potentially important sport-specific predictors were not included. Research shows that a child’s sporting experience is highly predictive of dropout, and the omission of sport-specific predictors limits the application of the study findings. It is currently unclear what the respective influence of sport-specific factors is when compared with socio-ecological predictors such as those presented in this study. Furthermore, while the large range of socio-ecological predictors included in the study is a strength, the validity and reliability of some measures used is unknown. In addition, parental or teacher report of some child variables such as physical activity level and gross motor coordination are subjective measures and may not provide a reliable measure of these important variables.

Future studies and interventions should consider participation and dropout through the lens of the Socio-Ecological Model [20]. In particular, attention should be payed to the interplay of child sex, sociocultural and socioeconomic indicators, and parental support. Given the consistency of studies in this area, culminating in these prospective and nationally-representative data, interventions to promote sports participation and prevent dropout from organised sports are urgently required and must be targeted to populations at highest risk of dropout and nonparticipation. These include girls, children of lower socioeconomic backgrounds, and children who receive low parental support. Preferential allocation should be given to children who meet all three criteria. Interventions should include multiple components and intervene at multiple levels of influence.

## Abbreviations

BMI: Body mass index; LSAC: Longitudinal study of Australian children; PA: Physical activity; PedsQL: Pediatric quality of life scale; SDQ: Strengths and difficulties questionnaire; VIF: Variance inflation factor.

## Competing interests

The authors have no competing interests to disclose.

## Authors’ contributions

SV conceptualised and designed the study, carried out the initial analyses, drafted the initial manuscript, and approved the final manuscript as submitted. DC conceptualised and designed the study, reviewed and revised the manuscript, and approved the final manuscript as submitted. AO conceptualised and designed the study, reviewed and revised the manuscript, and approved the final manuscript as submitted. All authors read and approved the final manuscript.

## Authors’ information

SV is a postdoctoral research fellow at the Early Start Research Institute, University of Wollongong. DC is a National Heart Foundation of Australia postdoctoral research fellow at the Early Start Research Institute, University of Wollongong. AO is a Professor at the Early Start Research Institute, University of Wollongong.
